# Dyspnea assessment and adverse events during sputum induction in COPD

**DOI:** 10.1186/1471-2466-6-17

**Published:** 2006-06-29

**Authors:** Demosthenes Makris, Nikolaos Tzanakis, Joanna Moschandreas, Nikolaos M Siafakas

**Affiliations:** 1 Department of Thoracic Medicine, University of Crete, Medical School, Heraklion, Greece

## Abstract

**Background:**

The inhalation of normal or hypertonic saline during sputum induction (SI) may act as an indirect bronchoconstrictive stimulus leading to dyspnea and lung function deterioration. Our aim was to assess dyspnea and adverse events in COPD patients who undergo SI following a safety protocol.

**Methods:**

Sputum was induced by normal and hypertonic (4.5%) saline solution in 65 patients with COPD of varying severity. In order to minimize saline-induced bronchoconstriction a protocol based on the European Respiratory Society sputum induction Task group report was followed. Dyspnea change was scored using the Borg scale and lung function was assessed by spirometry and oximetry.

**Results:**

Borg score changes [median(IQR) 1.5(0–2)] were observed during SI in 40 subjects; 16 patients required temporary discontinuation of the procedure due to dyspnea-general discomfort and 2 did not complete the session due to dyspnea-wheezing. The change in Borg dyspnea score was significantly correlated with oxygen saturation and heart rate changes and with discontinuation of the procedure due to undesired symptoms. 19 subjects presented an hyperresponsive reaction (decline>20% from baseline FEV_1_). No significant correlation between Borg changes and FEV_1_decline was found. Patients with advanced COPD presented significantly greater Borg and oxygen saturation changes than patients with less severe disease (p = 0.02 and p = 0.001, respectively). Baseline FEV_1_, oxygen saturation and 6MWT demonstrated significant diagnostic values in distinguishing subjects who develop an adverse physiologic reaction during the procedure.

**Conclusion:**

COPD patients undergoing SI following a safety protocol do not experience major adverse events. Dyspnea and oxygen desaturation is more likely to occur in patients with disease in advanced stages, leading to short discontinuation or less frequently to termination of the procedure. Baseline FEV_1_, oxygen saturation and 6MWT may have a prognostic value for the development of these adverse events and might be useful to be evaluated in advance.

## Background

Induced sputum examination is a relatively non invasive method standardized by Pin et al[1] as an alternative to bronchoscopy procedure for collecting secretions and inflammatory cells from the airways of subjects with Chronic Obstructive Pulmonary Disease (COPD) and bronchial asthma. The method consists of the induction of airways secretions after the inhalation of progressively increased concentrations of saline aerosol. It is more feasible than bronchoscopy and is considered to provide repeatable and valid results [2].

However, the inhalation of normal or hypertonic saline, may also act as an indirect bronchoconstrictive stimulus in subjects with airflow limitation, leading to further lung function deterioration and worsening of symptoms such as dyspnea[2]. Concerns about the safety of the procedure have been raised especially after the report of a fatal asthma attack, precipitated by inhalation of distilled water[3]. In asthma, the method is considered safe when standard guidelines are applied[4].

In COPD lung function deterioration has been reported during the procedure [5-7]. European Respiratory Society (ERS) sputum induction Task group report[5] has underlined the lack of systematic studies addressing safety issues in patients with advanced COPD. In previous studies [6-10] the main objective was mainly the lung function changes and forced expiratory volume in one second (FEV_1_) decline during SI but, little attention was paid in the worsening of dyspnea. However, dyspnea development during SI may affect the tolerability of the method in COPD[8,10]. Thus, for research purposes or for clinical studies investigating cells or mediators measurable in sputum, it is important to realize the potential danger imminent in this procedure and to improve the tolerability of SI.

In the present study we performed SI by administrating normal and hypertonic saline in a group of COPD patients, as part of a longitudinal study of lung function decline. Our primary aim was 1) to assess dyspnea and adverse events during the procedure and, 2) to evaluate the relation between dyspnea and lung function change or oxygen desaturation that may occur during the procedure. A secondary endpoint in this study was to determine the diagnostic value of baseline parameters in distinguishing patients who will experience an adverse reaction during SI.

## Methods

### Subjects

Sixty five patients, 21 current smokers and 44 ex-smokers, with stable COPD were recruited by consecutive sampling from a cohort of a longitudinal study of lung function decline in COPD and gave their consent to participate in the study. The diagnosis of COPD was based on the Global Initiative for Chronic Obstructive Lung Disease (GOLD) consensus criteria[11]. All patients with COPD had been free of an acute exacerbation for at least 4 weeks before the study and had not received antibiotics or corticosteroids (oral or inhaled) over the same period. No subject had a history of asthma or allergic rhinitis. Twenty patients had concomitant cardiovascular disease in stable condition. The hospital ethical committee ('Agios Georgios' Chania General Hospital, Crete, Greece) approved the protocol.

### Study design

Patients attended on two consecutive days. Short acting B_2 _agonists and/or anticholinergic inhalers were withheld for a minimum of 8 hours and long acting B_2 _agonists for 12 hours before each visit. The subjects were followed for the subsequent two weeks period in order to assign late adverse events.

### Baseline assessment (visit 1)

Baseline characteristics were evaluated[12] and 6MWT was performed according to standardized guidelines[13]. The 6MWT was performed indoors about the same time of day, along a 100-feet flat, straight, enclosed hallway with a hard surface that was seldom travelled. The walking course was 30 meters in length and it was marked every 3 meters. Instructions to patients were given according to accepted recommendations[13]. The patient should sit at rest in a chair, located near the starting position, for at least 10 minutes before the test started. Clothing and shoes should be appropriate for walking. During that time, oxygen saturation, pulse and blood pressure were measured and baseline dyspnea was assessed using the Borg scale. A physician should stand near the starting line during the test without walking with the patient. Only the standardized phrases for encouragement[13] were used during the test. When test was finished postwalk Borg dyspnea, oxygen saturation and pulse rate were recorded, as well as the total distance covered.

### Sputum induction (visit 2)

Sputum was induced via inhalation of saline solutions aerosol, generated by an ultrasonic nebulizer (Ultraneb 2000; DeVilbSIs; Somerset, PA) modifying a previously described protocol[14]. Aerosols of normal saline and hypertonic saline (NaCl 4.5%) were inhaled for subsequent periods of 2 and 8 minutes each (4 periods, 20 minutes in total). The sample was determined to be adequate if at least 1 ml of sputum was collected into a sterile container.

### Adverse events

The patients were free to ask for discontinuation of the procedure in case they experienced undesired symptoms. At the end of sputum induction, they were asked to record these symptoms in a chart. Any discontinuation of the procedure due to undesired symptoms was termed as "mild adverse event". Any discontinuation of the procedure due to symptoms requiring acute medical pharmaceutical intervention-hospitalization was termed as "major adverse event".

### Dyspnea assessment

Perception of dyspnea during SI was defined as sensation of bronchoconstriction or, chest tightness or, inability to take a deep breath or, sensation of effort to breath. Dyspnea intensity was rated on the Borg dyspnea scale[15] immediately before each lung function measurement and at the end of sputum induction.

### Lung function measurement and O_2 _desaturation

Spirometry was performed at baseline and at the end of each time period with a computerized system (Lab, 2.12; Jaeger; Wuerzburg, Germany). This system, which meets the ATS standards, was calibrated every day with standardized techniques according to guidelines[16]. Pulse oximetric saturation (SpO_2_) was recorded immediately before each FEV_1 _measurement using pulse oximetry (Nonin 8500 M; Nonin Medical; Minneapolis, MN).

### Safety protocol

In order to minimize the broncoconstrictive response to saline inhalation, a safety protocol was followed. The protocol was based on the European Respiratory Society sputum induction Task group report[5]:

a) All subjects were premedicated with 200 μg salbutamol via metered-dose inhaler 30 minutes before spirometry. b) If mild adverse events took place, clinical evaluation was carried out. c) If major adverse events or life threatening adverse events occurred there was a termination of the procedure and patients were treated appropriately. d) Bronchodilators were administered at the end of SI in any subject who experienced dyspnea or decline of FEV_1_>20% from the baseline.

The reproducibility of the above was assessed on a pilot study on 10 subjects. These subjects were chosen from the outpatient clinics of Chania General Hospital, Crete, Greece. Five of them were selected based on a physician's diagnosis of stable COPD. The five other subjects chosen from the list of non respiratory outpatient clinics did not have any respiratory disease and did not complain of dyspnea. They were scheduled for two visits, 2 weeks apart. They underwent SI and dyspnea was evaluated by the Borg scale. The reproducibility in this sample was 95%.

### Statistical analysis

Descriptive statistics were used to summarize the baseline characteristics and the results were expressed as means(SE) or stated otherwise. Normal distribution was assessed using the Kolmogorov-Smirnov Z test. The independent samples t-test was applied for the comparison of approximately normally-distributed variables and the Mann-Whitney U test where there was evidence of non-normality. Categorical variables were compared using the chi-square test. Borg score change (ΔBorg), adverse events were considered as outcome variables. Correlations between outcome variables and clinical or lung function variables were assessed appropriately either by Pearson's r^2 ^or by Spearman's rho. To determine the prognostic value of various parameters in distinguishing patients who will experience an adverse physiologic reaction (ΔBorg >0 and adverse events) during SI, receiver-operating-characteristic (ROC) curves were constructed to assign cut-off values and their diagnostic utility. A p value of less than 0.05 was considered as statistically significant. The statistical package SPSS 13.0, (Chicago, IL, USA) was used for the entire analysis.

## Results

Patient's baseline characteristics are demonstrated in Table 1. 60 subjects (92%) produced acceptable sputum with a mean (SE) percentage viability 96(9) and squamous cells contamination 17.5(12). All five subjects that did not provide sufficient sputum samples experienced adverse events during the procedure: 2 dyspnea (chest tightness) – excessive wheezing; 2 dyspnea (sensation of effort to breath) – general discomfort; 1 nausea. These subjects presented significantly greater ΔBorg [3(1) versus 1.5(1) p = 0.001], ΔSpO_2 _[-2.5(-2) versus -1(1) p = 0.001] and ΔFEV_1 _[-18(12) versus -10(8), p = 0.001], compared to all other patients. Unsuccessful session of sputum induction was more likely in the group of patients with COPD of stage IV than in patients with less severe disease [Relative Risk (RR) 18, 95%CI 1.7–80].

### Adverse events

No major event requiring hospitalization occurred during the procedure. 18 of the 65 patients presented 28 minor adverse events: 16 dyspnea-general discomfort, 9 general discomfort, 1 nausea; 2 did not complete the session due to dyspnea-excessive wheezing. One patient with COPD stage IV experienced a COPD exacerbation two days later treated adequately at home. Patients with stage IV COPD had a significantly increased risk to be in this subgroup compared to patients with less severe disease (RR 2.9, 95%CI 1.4–6).

### Borg score dyspnea

**Figure 1:**
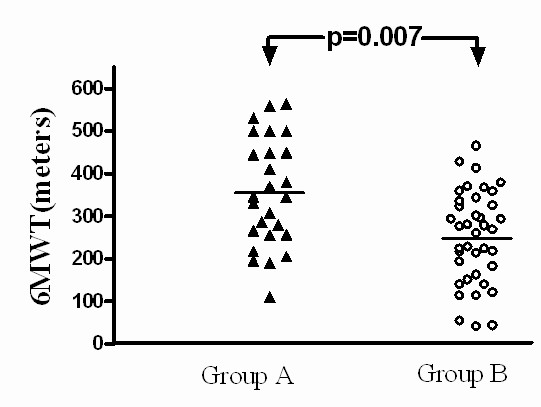
**6MWT distance in COPD patients according to Borg dyspnea score during sputum induction**. Group A represents patients with mean changes in Borg score = 0; Group B represents patients with mean changes in Borg score >0. Bars represent mean values.

**Figure 2:**
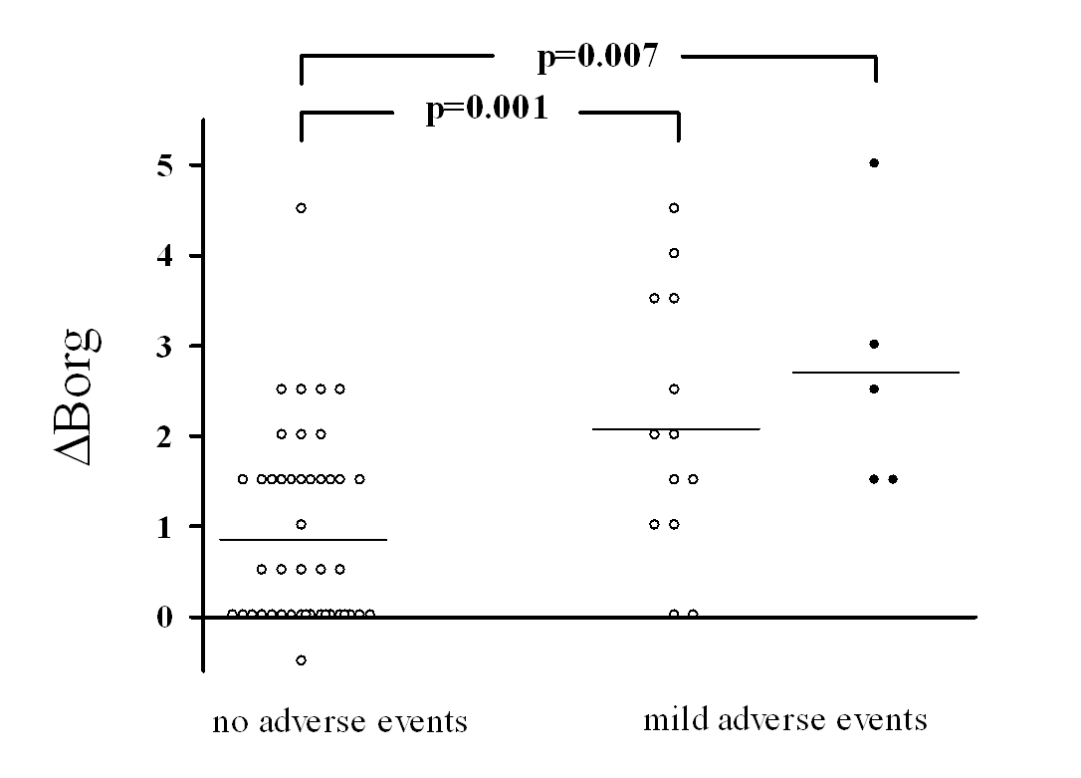
**Borg score changes in COPD patients during sputum induction, according to adverse events and success of procedure**. Individual values of average changes in Borg dyspnea scale during sputum induction. Mild adverse events were defined as discontinuations of the procedure due to undesired symptoms including dyspnea (sensation of bronchoconstriction, chest tightness, inability to take a deep breath, sensation of effort to breath), wheezing, nausea. Open circles represent patients that completed the procedure successfully; closed circles represent patients that did not complete the procedure successfully due to mild adverse events.

40 subjects (61%) demonstrated ΔBorg>0. These patients had relatively advanced disease (Table 2), lower baseline SpO_2 _[89(0.9) versus 93(0.4), p = 0.007], 6MWT (meters) [227(18) versus 352(26), p = 0.007] (Figure 1), compared to patients with ΔBorg = 0. Borg score changes were significantly greater in patients who experienced adverse events compared to the rest of the patients (Figure 2). There were no significant differences in ΔBorg between smokers and ex-smokers [1.4(0.5) versus 1.2(0.4), respectively p = 0.11].

### Lung function

**Figure 3:**
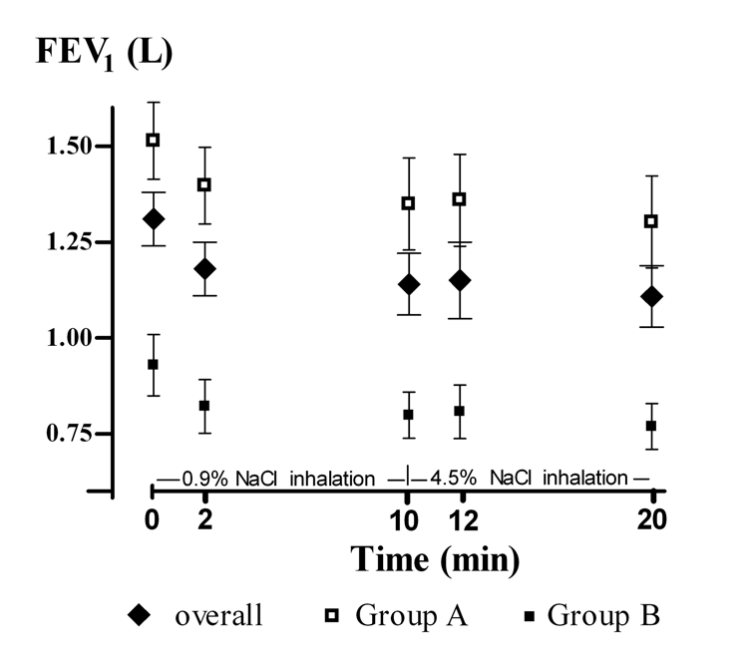
**FEV_1 _change during sputum induction**. Percentage change in FEV_1 _with standard errors during sputum induction. Group A represents patients with mean changes in Borg score ≤0; Group B represents patients with mean changes in Borg score >0.

The average decline of FEV_1_(ΔFEV_1_) during the procedure is demonstrated in Figure 3. The mean(SE) change in FEV_1 _was overall -9.9(2)% from the baseline. FEV_1 _had already fallen by 9.5(2)% from the baseline 2 minutes after saline inhalation. 19 subjects out of 61 (31%) presented an hyperresponsive reaction to saline inhalation (loss of > 20% from the baseline FEV_1_).

### Correlations between Borg score and oxygen desaturation, lung function

The average ΔBorg was significantly correlated with average ΔSpO_2_, (r = -0.38, p = 0.001) and adverse events (rho = 0.51, p = 0.01). Significant correlations were also present between the ΔBorg and ΔSpO_2 _when each time period was studied separately: 0 to 2 minutes, 2 to 10 minutes, 10 to 12 minutes and 12 to 20 minutes (r = -0.26, p = 0.01, r = -0.4, p = 0.002, r = -0.21, p = 0.01 and r = -0.45, p = 0.001, respectively).

In contrast there were no significant correlations between ΔBorg and ΔFEV_1 _either considering the average or the values in each time period.

### Diagnostics

Table 3 depicts the threshold values of various parameters in distinguishing patients that presented an adverse physiologic reaction during the procedure (defined as ΔBorg>0 + adverse events). Among them, 6MWT and MRC score demonstrated remarkable specificity with high positive and negative predictive values, while post-bronchodilation FEV_1 _demonstrated the highest sensitivity.

## Discussion

This prospective study represents the largest systematic report of adverse events and dyspnea evaluation during sputum induction in COPD. Our results suggest that COPD patients who undergo sputum induction, following a safety protocol, do not experience major adverse events. However, the patients may have an increase perception of dyspnea [overall increase in Borg score median[(IQR) 1.5(0–2)] and desaturation and may require short discontinuation of the procedure due to undesired symptoms. We found that dyspnea changes during sputum induction were significantly correlated with oxygen saturation, heart rate changes and mild adverse events. This adverse physiologic reaction was more frequent in subjects with advanced COPD. Notably, patients of stage IV in GOLD staging of severity, presented an increase risk to have unsuccessful sputum induction and to have minor adverse events during the procedure compared to patients with less severe disease (RR 2.9, 95%CI 1.4–6). In addition we found that the baseline values of post-bronchodilation FEV_1_, of oxygen saturation and of 6MWT have a diagnostic value in distinguishing patients who develop an adverse reaction during SI. To the best of our knowledge this has not been reported.

In previous studies addressing SI safety in COPD, the measurement of FEV_1 _was considered enough to diagnose acute lung responses[6,7,9]. However, an excessive fall in FEV_1 _during SI is not always associated with clinical deterioration[9] or dyspnea development[6]. Thus, it is not unlikely that assessing only FEV_1 _during the procedure, early signs of clinical deterioration may be undetected. Consequently, the development of adverse events may affect the tolerability of the procedure[8,9]. In our study, special attention was paid to evaluate dyspnea, in addition to FEV_1 _assessment. We found that patients undergoing SI may experience an adverse physiologic reaction, characterized by worsening of dyspnea, undesired symptoms and oxygen desaturation. This adverse reaction was not significantly related to FEV_1 _decline but affected the tolerability of the procedure in a proportion of patients. This may have importance in research or clinical studies, especially in patients with advanced disease.

In the studied population, we included subjects with advanced disease since European Respiratory Society report has underlined the lack of systematic studies in this category of patients[5]. Normal and hypertonic saline was administered even in subjects with very severe disease. We found that COPD patients experienced significant Borg score and oxygen saturation changes, associated with disease severity. COPD subjects of stage IV had an increased risk of developing dyspnea, requiring subsequently discontinuation of the procedure. The degree of discomfort led eight out of 14 (57%) patients in this category to temporary discontinuation and two of them (14%) to early termination of the procedure. In addition, 4 out of 5 patients who did not provide sufficient sputum sample, had stage IV COPD. Hence, patients with advanced COPD may experience excessive dyspnea during sputum induction and they might be reluctant to repeat the procedure in the future. Therefore, sputum induction in this category of patients must be performed with great caution and in the ground of our findings careful monitoring of oxygen saturation and dyspnea is essential.

In the present study all subjects were premedicated with salbutamol. B_2_-agonists and anticholinergic inhalers were withheld before SI in order to standardize further our assessment. It is known that inhaled salbutamol does not provide full protection from bronchoconstriction as it has been demonstrated by the adverse responses after saline inhalation[7,9]. However, it remains unclear whether the magnitude of bronchoconstriction could be prevented by pretreatment with larger doses of inhaled salbutamol or with another type of bronchodilator or antinflammatory treatment[9,10]. A prospective study is necessary to test this hypothesis.

In this study, 31% of the patients demonstrated an excessive fall (>20%) of FEV_1_. According to previous reports, excessive FEV_1 _decline ranges, between 11[6] and 50%[9], depending on the COPD population studied. Interestingly, we found that FEV_1 _decline had almost reached the average decline 2 minutes after saline inhalation and that FEV_1 _did not return to baseline during the procedure. In previous studies in COPD patients[8-10], there hasn't been any assessment before the 5^th ^minute following saline inhalation. In these studies, the greatest decline in FEV_1 _seems to occur constantly at the beginning of the procedure, following similar time course patterns to our assessments. The time course pattern of FEV_1 _could be explained by the underlying mechanism of the bronchoconstrictive response. The inhalation of normal or hypertonic saline may trigger mast cell and basophil degranulation, in response to an increase of airway osmolarity[17-20]. The release of bonchoconstrictive mediators from mast cells is rapid and essentially completed by five minutes[21]. In line with this early inflammatory response, it has been reported in a time course assessment study in asthmatic patients[18], that the maximal mean fall in FEV_1 _occurs at 3 minutes post saline inhalation. In this ground, premedication of the patients with bronchodilators should be a standard safety measure of the procedure. Future studies of different design may identify which is the most effective bronchodilator to prevent this bronchoconstrictive response.

An interesting point in our study is that the development of dyspnea and the fall of the FEV_1 _were not significantly correlated. One would expect patients with the most severe airway obstruction to be the most dyspnoeic. However, some patients with severe airway obstruction are minimally symptomatic, whereas others with little objective dysfunction appear to be very dyspnoeic[22]. Several studies have investigated the correlation between dyspnea and lung function[23]. Mahler et al reported that dyspnea and baseline pulmonary function are independent quantities in patients with COPD[24]. Subsequent studies employing newer techniques to quantify breathlessness found either no significant or weak correlations with FEV_1_[25,26]. Thus, an excessive fall in FEV_1 _is not always correlated with symptoms development and with dyspnea scale scores in COPD[6,9].

A reasonable explanation for this discrepancy between FEV_1 _decline and clinical deterioration during SI, may be the subjectiveness of dyspnea perception[27]. Unlike asthmatic patients who experience episodic bronchoconstriction, those with COPD demonstrate chronic airflow limitation that might lead to desensitization. Ottanelli and colleagues have previously reported that a reduced perception of dyspnea during bronchoconstriction may be present in COPD patients[28]. A reduced perception of dyspnea might delay self referral or lead to underreport of discomfort during the procedure. In addition, it may be that some dyspnoeic patients in our study, did not develop their "potential maximal" drop in FEV_1_, because they felt discomfortably and interrupted the procedure thus, demonstrating a submaximal effort in lung fuction testing. Furthermore, dyspnea during a bronchoconstrictive challenge is associated not only to airway obstruction but also to hyperinflation[29]. In fact, dyspnea perception may be better related with acute hyperinflation than with airflow obstruction sensation in patients with chronic airflow obstruction[28]. Patients with advanced COPD may develop dynamic hyperinflation in the setting of a bronchocontrictive stimulus[9,29]. In the present study the most severely affected patients in terms of baseline disease severity presented the greatest perception of dyspnea during SI. Thus, it is likely that a proportion of patients may have experienced dyspnea during SI due to acute hyperinflation.

In this ground other lung function parameters may be considered in addition to FEV_1_, when addressing safety in SI. It has been demonstrated that forced inspiratory rather than expiratory parameters were more sensitive in detecting SI related lung function deterioration and were better associated to dyspnea[9,30]. Forced inspiratory volume in one second(FIV_1_) is less affected by airway collapse than FEV_1_, reflecting obstruction and hyperinflation[9]. In addition, acute inspiratory capacity changes (IC) account in part for the variability in the perception of dyspnoea after accounting for changes in FEV_1 _during bronchoconstriction in patients with chronic airflow obstruction[28]. These data, along with the disassociation between dyspnea and FEV_1 _in our study, suggest that possibly other parameters, like FIV_1 _or IC should be brought forward to monitor lung function deterioration and adverse events development, during SI. In this ground a new insight for the reason of dyspnea during SI might also be provided. However, the present study was designed to assess dyspnea intensity and adverse events during SI based on ERS sputum induction task group report [5] and thus other lung function parameters were not assessed.

In the present investigation, ΔBorg was significantly correlated with ΔSpO_2_. However the correlations between dyspnoea intensity and oxygen saturation changes were weak. A plausible explanation may be that the relationship between hypoxia – ventilatory response and breathlessness in patients with COPD is not linear. Thus, the level of breathlessness is related to hypoxaemia but not in all levels of desaturation[31]. In addition, dyspnea sensation may result from pathophysiological abnormalities that can be related to non respiratory mechanisms[32]. Therefore, the weak correlations between dyspnea and oxygen saturation changes could be attributable to other factors (emotional, cognitive) which have not been evaluated in the present study.

In the present study, we evaluated the diagnostic performance of baseline clinical characteristics of the patients undergoing SI, in order to distinguish those who will develop an adverse physiologic response during SI. This is important because clinical parameters which can be measured in a simple way, before performing the test, give useful information in advance. In addition, predictors of adverse events and lung function deterioration during SI are not yet widely known [6,9,27]. We found that the development of dyspnoeic events during SI, could be better predicted by the post-bronchodilation FEV_1_(%pred), the MRC score, the oxygen saturation and the 6MWT. To the best of our knowledge, this has not been reported until now[5,7,10].

MRC score is a good predictor of exercise capacity. It has showed a consistent relationship with Borg rating and a significant correlation with breathlessness and dynamic hyperinflation measured during walking[33]. Baseline oxygen saturation is also reported to be associated with the hypoxemia during inhalation provocative tests[34]. In addition, our investigation showed that COPD patients with good performance status, by means of walking more than 155 meters during 6MWT, will be less prone to develop dyspnea during the procedure. This is likely to be due to dynamic hyperinflation. 6MWT performance is associated to the oxygen uptake, to the severity of chronic dyspnea in COPD patients and it may also be related with the dynamic hyperinflation which is developed in patients after certain stimuli[9,35]. We believe that since subjects with moderate to severe COPD are characterized by hyperinflation and low performance, 6MWT is possibly a good predictor of developing dyspnea after a stimulus such as the inhalation of saline [9,22].

In summary, we found that normal and hypertonic saline-induced sputum is a safe technique, when certain precautions are taken, in patients with COPD. It is safe even for patients in an advanced stage of the disease. However, excessive dyspnea is more likely to occur in these patients, leading in temporary or permanent discontinuation, affecting tolerance and success of the procedure. Therefore, sputum induction must be performed with great caution and careful monitoring of dyspnea and oxygen saturation in patients with very severe COPD. Post-bronchodilation FEV_1_(%pred), oxygen saturation and 6MWT have a prognostic value for the development of dyspnea during SI and it would be useful to be evaluated in advance. We believe that this is important information and favors further the improvement of SI safety and tolerance especially in advanced COPD.

## Abbreviations

COPD = Chronic Obstructive Pulmonary Disease

FIV_1 _= forced inspiratory volume in one second

FEV_1 _= forced expiratory volume in one second

GOLD = Global Initiative for Chronic Obstructive Lung Disease

IC = Inspiratory Capacity

MRC = Medical Research Council

SI = sputum induction

ΔBorg = Borg score changes

ΔFEV_1 _= FEV_1 _changes

ΔBorg_6MWT _= Borg dyspnea score changes after 6MWT;

6MWT = six minutes walking test.

## Competing interests

The author(s) declare that they have no competing interests.

## Authors' contributions

DM participated in the data collection and drafted the manuscript. NT participated in the data collection, design and coordination of the study. JM performed the statistical analysis. NMS participated in the design of the study and revised the article for important intellectual content. All authors read and approved the final manuscript.

## Pre-publication history

The pre-publication history for this paper can be accessed here:



## Figures and Tables

**Table 1:** Baseline characteristics of all 65 COPD patients

Age, yrs	66(1)
M/F	60(92)/5(8)
Current/ex smokers	21(32)/44(68)
Pack years	53(3.9)
FEV_1 _(% predicted)	48(2.4)
FEV_1_/FVC	0.46(0.07)
Oxygen saturation %	92(0.4)
	
MRC dyspnea score	1 (0–4)
Chronic cough	44(6)
Chronic sputum	31(47)
Chronic wheeze	21(32)
Inhaled long acting b-agonists	41(63)
Inhaled corticosteroids	31(47)
	
6MWT, meters	311(18)
ΔBorg_6MWT_	2.1(0.2)

Continuous data are expressed as mean (SE), categorical data are expressed as n (%), MRC score is expressed as median (range).

**Table 2:** Lung function, pulse oximetry and Borg dyspnea score during sputum induction, according to COPD severity.

	**Overall**	**COPD severity (GOLD stages)**				
		**Stage I**	**Stage II**	**Stage III**	**Stage IV**	
			
	n = 65	n = 5	n = 24	n = 22	n = 14	
		
Median (IQR) change*						*p-value*
						
**Borg**	1.5 (0, -2)	0 (0, 1)	1 (0, 1)	1.5 (0, 2)	1.5 (0, 3)	0.02
**SpO_2 _%**	-1 (0, -2)	-0 (0, 0)	-1 (0, -1.5)	-1.5 (0, -3)	-2.5 (-1.5, -3)	0.001
**Heart rate**	25 (10, 33)	15 (8, 22)	17 (10, 25)	21 (12, 29)	32(10, 45)	0.09
**FEV_1_**	-9.5 (-3, -15)	-11 (-3, -17)	-5 (0,-8)	-12 (-3, -17)	-9 (-3, -12)	0.32

* change from the post-bronchodilation baseline value

**Table 3:** Diagnostic performance of baseline characteristics of COPD patients in distinguishing those with an adverse reaction during SI.

	**Cut-off value**	**Sensitivity**	**Specificity**	**Positive predictive value**	**Negative predictive value**
	
**A^2^ge**	>70	65	52	42	77
**FEV_1_, (% pred)**	<40	83	64	47	91
**ΔSpO_2 _(%)**	≤91	74	78	54	88
**MRC score**	> 2	52	86	56	82
**Borg**	>4	66	55	44	80
**6MWT distance (m)**	≤155	52	98	90	84
**ΔBorg_6MWT_**	>2	82	55	33	73

FEV_1 _= post-bronchodilation forced expiratory volume in one second; ΔSpO_2 _= oxygen saturation average changes; MRC = Medical Research Council; 6MWT = six minutes walking test; ΔBorg_6MWT _= Borg dyspnea score change after 6MWT.
